# Enhancing Winter Wheat Soil–Plant Analysis Development Value Prediction through Evaluating Unmanned Aerial Vehicle Flight Altitudes, Predictor Variable Combinations, and Machine Learning Algorithms

**DOI:** 10.3390/plants13141926

**Published:** 2024-07-12

**Authors:** Jianjun Wang, Quan Yin, Lige Cao, Yuting Zhang, Weilong Li, Weiling Wang, Guisheng Zhou, Zhongyang Huo

**Affiliations:** 1 Jiangsu Key Laboratory of Crop Genetics and Physiology/Jiangsu Key Laboratory of Crop Cultivation and Physiology, Agricultural College of Yangzhou University, Yangzhou 225009, China; wangjianjun@yzu.edu.cn (J.W.); mx120220725@stu.yzu.edu.cn (Q.Y.); mz120221323@stu.yzu.edu.cn (Y.Z.); mx120220735@stu.yzu.edu.cn (W.L.); 007465@yzu.edu.cn (W.W.); 2 Jiangsu Co-Innovation Center for Modern Production Technology of Grain Crops, Yangzhou University, Yangzhou 225009, China; 3 College of Life and Health Sciences, Anhui Science and Technology University, Chuzhou 233100, China; 1338220301@ahstu.edu.cn; 4 Joint International Research Laboratory of Agriculture and Agricultural Product Safety, Yangzhou University, Yangzhou 225009, China; gszhou@yzu.edu.cn

**Keywords:** winter wheat, SPAD value, vegetation indices, texture indices, discrete wavelet transform, machine learning, flight altitudes

## Abstract

Monitoring winter wheat Soil–Plant Analysis Development (SPAD) values using Unmanned Aerial Vehicles (UAVs) is an effective and non-destructive method. However, predicting SPAD values during the booting stage is less accurate than other growth stages. Existing research on UAV-based SPAD value prediction has mainly focused on low-altitude flights of 10–30 m, neglecting the potential benefits of higher-altitude flights. The study evaluates predictions of winter wheat SPAD values during the booting stage using Vegetation Indices (VIs) from UAV images at five different altitudes (i.e., 20, 40, 60, 80, 100, and 120 m, respectively, using a DJI P4-Multispectral UAV as an example, with a resolution from 1.06 to 6.35 cm/pixel). Additionally, we compare the predictive performance using various predictor variables (VIs, Texture Indices (TIs), Discrete Wavelet Transform (DWT)) individually and in combination. Four machine learning algorithms (Ridge, Random Forest, Support Vector Regression, and Back Propagation Neural Network) are employed. The results demonstrate a comparable prediction performance between using UAV images at 120 m (with a resolution of 6.35 cm/pixel) and using the images at 20 m (with a resolution of 1.06 cm/pixel). This finding significantly improves the efficiency of UAV monitoring since flying UAVs at higher altitudes results in greater coverage, thus reducing the time needed for scouting when using the same heading overlap and side overlap rates. The overall trend in prediction accuracy is as follows: VIs + TIs + DWT > VIs + TIs > VIs + DWT > TIs + DWT > TIs > VIs > DWT. The VIs + TIs + DWT set obtains frequency information (DWT), compensating for the limitations of the VIs + TIs set. This study enhances the effectiveness of using UAVs in agricultural research and practices.

## 1. Introduction

The SPAD (Soil–Plant Analysis Development) value represents the relative chlorophyll content and is significant in crop cultivation and breeding to evaluate crops’ photosynthetic capacity and nutritional health. It provides important indicators for rapid fertilization diagnoses and crop variety screenings [1,2,3]. Winter wheat is one of the vital staple crops in China, and is essential for maintaining national food security and driving economic development [4]. Therefore, the accurate and efficient monitoring of winter wheat SPAD values holds immense importance.

Advancements in remote sensing (RS) technology have led to numerous studies confirming that monitoring winter wheat SPAD values through RS is the most effective and non-destructive method available [5,6,7]. In particular, optical sensors carried by unmanned aerial vehicles (UAVs) can obtain RS images with a fine spatial (cm level) and spectral resolution. They can adjust flight altitude and coverage area according to specific requirements, providing detailed spectral and spatial information on winter wheat [8]. When using a fixed focal length for UAV image acquisition, the spatial resolution decreases with the increase in UAV flight altitude. Researchers generally believe that a higher spatial resolution usually means more detailed information on winter wheat growth [9,10]. Therefore, when obtaining UAV images, there is a tendency to lower the flight altitude as much as possible [11,12,13]. For example, the widely used DJI Phantom 4 multispectral UAV (DJI, Inc., Shenzhen, China) typically sets the flight altitude at 10–30 m (with a resolution of 0.52–1.59 cm/pixel) in studies predicting winter wheat SPAD values [14,15,16]. However, a higher spatial resolution requires lower UAV flight altitude, often resulting in a longer image acquisition time.

At present, UAVs’ primary limitation is the battery capacity and net weight, as a high battery capacity and limited battery net weight result in increased flight duration [17]. An ordinary UAV can usually operate safely for about 10–20 min, and recharging becomes necessary if the battery’s charge drops below 10% [18]. Since UAVs typically need to hover to capture images, a lower flight altitude means more hovering points, significantly increasing the flight time and making it difficult to collect more field images with limited batteries. Moreover, a longer flight time increases the likelihood of encountering lighting changes. While the utilization of UAVs for crop monitoring is growing swiftly, a significant challenge arises due to the varying illumination caused by fluctuating solar radiation and cloud cover. The incident spectral irradiance captured by UAV-mounted sensors blends plant properties and solar spectral irradiance. Consequently, image data acquired under variable illumination can yield misleading crop information [19]. For example, vegetation indices (VIs) derived from UAV images for crop monitoring and phenotyping can be affected by these variations. Discrepancies observed in these image data may stem from genuine crop variability or changing lighting conditions. Although certain VIs are less affected by brightness, they are inadequate for handling variable sunlight, given that cloud cover alters brightness and modifies the illumination’s spectral attributes.

Previous studies have begun to explore whether crop parameters can be effectively predicted at higher flight altitudes. For example, Xu et al. [20] collected original images at the flight altitude of 200 m, employing a DJI M600Pro UAV equipped with a Rikola hyperspectral camera (Senop Ltd., Oulu, Finland). They resampled original images at multiple spatial resolutions (26, 39, 52, 65, 78, 91, and 100 cm/pixel) to simulate images collected at various higher flight altitudes, exploring the “appropriate monitoring scale domain” for predicting above-ground biomass (AGB) of rice. However, predicting SPAD values (physiological parameters of crops) at different flight altitudes obviously differs from predicting AGB (morphological characteristics of crops) at different flight altitudes. More importantly, according to the “Interim Regulations on the Management of Unmanned Aircraft Flights”, implemented in China in 1 January 2024, the maximum altitude in light and small flight areas is capped at 120 m [21]. The study by Xu et al. [20] on original images collected at a UAV flight altitude of 200 m seems to lack practical value within China. Therefore, the current study should explore the highest possible flight altitude within 120 m (using a DJI P4-Multispectral UAV as an example, with a resolution of 1.06 cm/pixel) that can accurately estimate the SPAD values of winter wheat. This will facilitate rapid fertilization diagnosis in large-scale farmland and efficient variety selection in breeding fields with a large number of experimental plots.

Moreover, the booting stage is a stage where the vegetative and reproductive growth of winter wheat occur simultaneously, exerting the most significant influence on final yield and quality [22]. In the Yangtze River’s middle and lower reaches and the Huang-Huai-Hai Plain in China, the sowing period of winter wheat usually occurs from mid-September to late November, the tillering stage typically occurs from early December to early March of the following year (Duration ≈ 100 days), the jointing–booting stage usually occurs from mid-March to early April (Duration ≈ 30 days), the heading stage typically occurs from mid-April to early May (Duration ≈ 25 days), and the maturity stage usually occurs from early May to late May (Duration ≈ 20 days) [23,24]. The booting stage of winter wheat typically occurs in late March or early April. This stage is the peak photosynthesis and nutrient absorption period in winter wheat. Plants require a lot of nutrients and water to support their growth and development, as well as the formation of spikes and grains. During the booting stage, winter wheat has weaker resistance to adversity, and drought, high temperatures, pests, and diseases can significantly affect growth, development, and yield formation [25]. Therefore, the timely and efficient monitoring of SPAD values during winter wheat booting is crucial to ensuring final yield. In previous studies, spectral indices comprised of linear or nonlinear combinations of spectral reflectances at various bands were the most commonly used method for predicting SPAD values during the wheat booting stage [22,26]. However, several studies have reported that the accuracy of SPAD value prediction during the winter wheat booting stage is lower than predictions during other growth stages [27,28]. Yin et al. [16] concluded that, compared to the other growth stages, the model developed for predicting winter wheat booting stage SPAD values exhibits underestimation issues. Wang et al. [26] reported that the accuracy of SPAD value prediction varied significantly across growth stages, with the accuracy improving in the following sequence: booting stage < heading stage < milk filling stage < flowering stage.

Optical RS, as a passive RS method, often faces saturation and insufficient sensitivity issues when using VIs to predict SPAD values in the reproductive growth stage of winter wheat [29]. Moreover, spectral heterogeneity, where weak plants within high-density areas and strong plants within low-density areas exhibit similar spectral characteristics, further restricts the efficacy of VIs [30]. Therefore, predicting SPAD values during the winter wheat booting stage (a stage where nutritional and reproductive growth occur simultaneously) using VIs may lead to significant uncertainty. To overcome the limitations of VIs, researchers have begun to explore the potential of texture indices (TIs) in predicting the SPAD values of winter wheat. TIs describe the variability between target pixels and their neighboring pixels, offering insights into vegetation’s spatial dimension and reflecting the canopy structure. TIs improve the ability to detect subtle changes in canopy structure compared to VIs. Yin et al. [16] demonstrated the potential of TIs in predicting the SPAD values of winter wheat. Additionally, the fusion of VIs and TIs can improve the accuracy of the estimated SPAD values of winter wheat during the booting stage compared to using VIs or TIs alone. Nevertheless, the improvement in SPAD value predictions during the winter wheat booting stage obtained through the fusion of VIs and TIs is still limited.

VIs convey the spectral characteristics of RS images, whereas TIs capture the spatial information within RS images. Wavelet variables obtained through discrete wavelet transform (DWT) capture the frequency and spectral details within RS images to some extent [31,32], thereby compensating for the limitations associated with using solely spectral or spatial variables. This is one of the reasons why we attempt to introduce wavelet variables to predict SPAD values during the winter wheat booting stage. DWT is an effective signal-processing technique that decomposes the original spectral signal into low-frequency and high-frequency signals [33,34,35], effectively separating useful information from noise and using existing information [36]. The extensive literature searches we conducted indicate that there is currently no research using DWT to predict crop SPAD values remotely.

In summary, this study aims to (1) assess whether higher flight altitudes (40 to 120 m, using a DJI P4-Multispectral UAV as an example, with a resolution from 2.12 to 6.35 cm/pixel) can accurately predict SPAD values during the winter wheat booting stage compared to a baseline altitude of 20 m (using a DJI P4-Multispectral UAV as an example, with a resolution of 1.06 cm/pixel); (2) assess the different potentials of VIs, TIs, and DWT in predicting SPAD values during the winter wheat booting stage; and (3) assess whether various combinations of predictor variables (VIs + DWT, TIs + DWT, VIs + TIs, and VIs + TIs + DWT) can enhance the prediction of SPAD values during the winter wheat booting stage.

## 2. Materials and Methods

### 2.1. Study Site and Experiment Design

**Figure 1:**
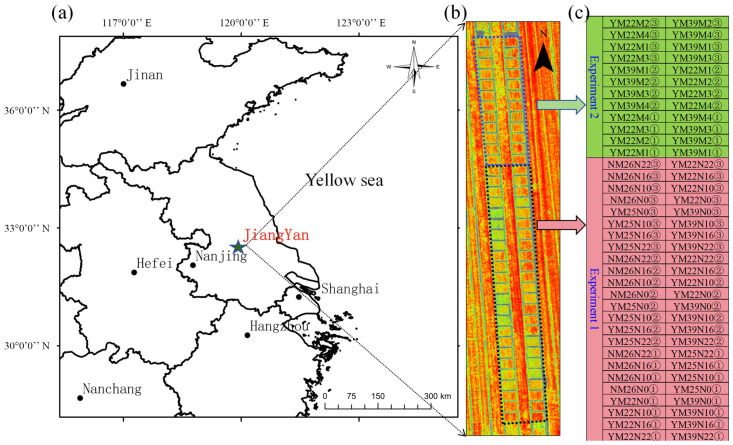
Study site and experimental design: (**a**) the geographical location of Jiangyan; (**b**) the NDVI image of the experimental field on 11 April 2023; (**c**) distribution of the plots and the various treatments. Note: N0 (0 kg/mu), N10 (10 kg/mu), N16 (16 kg/mu), and N22 (22 kg/mu) correspond to nitrogen fertilizer application rates of 0 kg/ha, 150 kg/ha, 240 kg/ha, and 330 kg/ha, respectively.

The one cultivation period experiment was carried out at the Jingxian Farm in Jiangyan District, Taizhou City, Jiangsu Province, China (32°34′23.43′′ N, 120°5′25.80′′ E) during the winter wheat cultivation period of 2022–2023 (Figure 1). The experimental site is situated in a rice–wheat rotation zone within the Yangtze River’s middle and lower reaches, characterized by a subtropical climate. This region’s annual average rainfall and temperature are approximately 1185.7 mm and 16.7 °C, respectively.

Experiment 1 involved four winter wheat varieties: Yangmai22 (YM22), Yangmai25 (YM25), Yangmai39 (YM39), and Ningmai26 (NM26). Each variety included four nitrogen treatment groups: a control group (0 kg/ha, N0) and treatment groups with nitrogen application rates of 150 kg/ha (N10), 240 kg/ha (N16), and 330 kg/ha (N22). Based on the growth stage of the winter wheat, the nitrogen application regime was divided into basal fertilizer, tillering fertilizer, jointing fertilizer, and booting fertilizer in a ratio of 5:1:2:2. The experiment utilized a split-plot design, with main plots corresponding to four nitrogen application rates treatments and subplots corresponding to four winter wheat varieties. Each experimental plot was replicated three times, totaling 48 plots.

**Figure 2:**
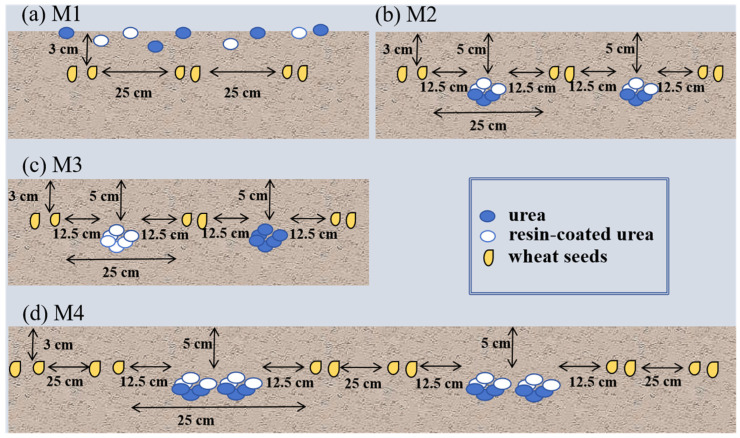
Four different nitrogen application methods (Experiment 2): (**a**) M1; (**b**) M2; (**c**) M3; (**d**) M4. Note: M1, M2, M3, and M4 each consist of 16 seed furrows, with a row spacing of 25 cm and a seeding depth of 3 cm. For M2, M3, and M4, the fertilizer application depth is 5 cm. M2 includes 17 fertilizer furrows, where resin-coated urea and urea are applied as a mixed strip between rows, with a fertilizer application depth of 5 cm and a seeding-to-fertilizer distance of 12.5 cm. M3 includes 17 fertilizer furrows, with resin-coated urea and urea applied separately between rows. M4 comprises eight fertilizer furrows, with resin-coated urea and urea applied as a mixed strip within the inter-row spaces.

Experiment 2 involved two winter wheat varieties: YM22 and YM39. Each variety was subjected to four different nitrogen application methods (Figure 2): broadcasting (M1), furrow application (M2), and two types of spaced furrow application (M3 and M4). Both urea and resin-coated urea were used as nitrogen fertilizers at a rate of 240 kg/ha. The experiment also used a split-plot design, with the varieties as the main plots and fertilizer types as the subplots. Like Experiment 1, each combination was replicated three times, totaling 24 plots.

In total, the experimental field was divided into 72 plots. The first 48 plots (starting from the south side of the experimental area) were assigned to Experiment 1, and the subsequent 24 plots were assigned to Experiment 2. Phosphorus (P_2_O_5_) and potassium (K_2_O) fertilizers were applied at a rate of 135 kg/ha each as basal fertilizer. Each plot was manually furrowed for sowing with a row spacing of 25 cm, covering an area of 12 m^2^ per plot. The sowing date was 8 November 2022. The basic seedling density was 240 × 10^8^ plants/ha (28.8 × 10^8^ plants/12 m^2^ plot).

### 2.2. Data Acquisition and Processing

#### 2.2.1. UAV Images Acquisition and Preprocessing

The research used the DJI P4-Multispectral UAV to acquire multispectral RS data during the winter wheat booting stage, capturing spectral bands: red band (R): 650 nm ± 16 nm; green band (G): 560 nm ± 16 nm; blue band (B): 450 nm ± 16 nm; near-infrared band (NIR): 840 nm ± 26 nm; Rededge (RE): 730 nm ± 16 nm. The data collection occurred at noon on 11 April 2023 (155 days after sowing (DAS)) under stable lighting conditions.

We employed the DJI GS Pro 2.0 iOS app (DJI, Inc., Shenzhen, China) to plan UAV flight missions and capture spectral images along predefined flight paths. Concurrently, high-definition RGB image data were synchronized. The UAV flew at an altitude of 20 m (with a resolution of 1.06 cm/pixel), with the sensor lens oriented vertically downward and an overlap rate of 80% for both heading and side views, with a flight duration of 39 min. The collected radiometric calibration panels and multispectral images of the winter wheat booting stage were imported into DJI Terra 2.3 software (DJI, Inc., Shenzhen, China) for image processing, resulting in the synthesis of original UAV images (20 m) for the winter wheat booting stage.

Subsequently, the original images with a flight altitude of 20 m (with a resolution of 1.06 cm/pixel) were resampled to multiple spatial resolutions in ENVI 5.6 software (ITT Exelis; Boulder, CO, USA) to simulate RS images captured at multiple UAV flight altitudes. The resampling was performed using the nearest neighbor algorithm, which selects the nearest pixel values to interpolate the original pixels to multiple sizes, ensuring grayscale recombination within the image [37].

In resampling, nearest neighbor interpolation assigns the pixel values of each point in the target image to the closest points in the source image, ensuring that mixed pixels are not generated [38]. This method does not modify the numerical value of the pixels, referred to as the digital number, and is widely used for resampling because of the speed with which it can be implemented and its sheer simplicity [39,40]. The study resampled the original UAV images to resolutions corresponding to flight altitudes of 40 m (with a resolution of 2.12 cm/pixel), 60 m (with a resolution of 3.18 cm/pixel), 80 m (with a resolution of 4.23 cm/pixel), 100 m (with a resolution of 5.29 cm/pixel), and 120 m (with a resolution of 6.35 cm/pixel). This was performed to align with the regulations of China’s Interim Measures for the Management of Unmanned Aircraft Flights [21], which came into effect on 1 January 2024, setting the upper limit for flights in light and small airspaces at a true altitude of 120 m.

#### 2.2.2. In Situ Wheat SPAD Measurements

During UAV data collection, simultaneous field measurements of SPAD values were conducted on 11 April 2023. We employed the SPAD-502Plus handheld chlorophyll meter (Konica Minolta, Tokyo, Japan) to measure 72 plots within the study area. The main specifications of the SPAD-502Plus handheld chlorophyll meter can be found in Table 1 [41]. The maximum temperature on the day of field SPAD value data collection was 27 °C, the minimum temperature was 11 °C, and there was no condensation, complying with the usage specifications of the SPAD-502Plus handheld chlorophyll meter, ensuring accurate SPAD value data acquisition.

A five-point sampling method was employed within each plot. One sampling point was located at the center of the plot, and the remaining four points were positioned near the four corners of the plot. At each sampling point, 10 flag leaves were randomly selected, resulting in a total of 50 flag leaves per plot. SPAD values were measured at three evenly spaced points (top, middle, and base portions of the flag leaf) on each leaf using the SPAD-502Plus handheld chlorophyll meter. Each flag leaf was measured three times, avoiding the leaf stem. Subsequently, the average SPAD value of the 50 flag leaves was calculated as the field-measured SPAD value for that plot.

### 2.3. Acquisition of RS Variables

#### 2.3.1. Selection of VIs

VIs amalgamate variations in reflectance across various wavelengths, thereby partially mitigating the impact of background factors on vegetation spectral properties. This process enhances the precision of expressing SPAD values using RS data [42]. In this experiment, the spectral reflectance (R, G, B, NIR, Rededge) of 72 plots was extracted in ENVI 5.6 software, and VIs (Table 2) were constructed through linear or nonlinear combinations.

#### 2.3.2. Extraction of TIs

The Gray-Level Co-occurrence Matrix (GLCM), reported by Haralick in 1973 [56], stands out as the most widely adopted texture extraction method. Its popularity stems from variables like rotation invariance, multiscale applicability, and computational efficiency [57]. Our study extracted eight GLCM-TIs from the original spectral band images of UAV multispectral data in ENVI. The extraction process employed a window size of 7 × 7 and a direction of (2, 2), generating 40 TIs calculated across all original spectral bands.

#### 2.3.3. Extraction of DWT

DWT is a signal processing technique that decomposes a signal into frequency components of varying scales [58]. Unlike traditional transform methods such as Fourier Transform, DWT provides both time and frequency domain information simultaneously, making it advantageous in processing non-stationary signals and extracting local variables.

**Figure 3:**
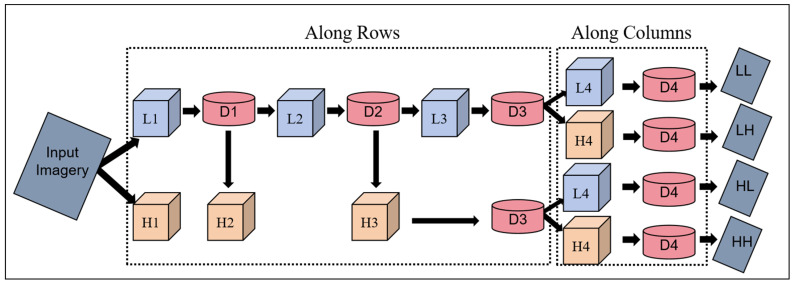
Decomposition process using the bior 1.3 wavelet basis function for DWT. Note: L1 represents the first low-pass filter. L2 represents the second low-pass filter. L3 represents the third low-pass filter. L4 represents the fourth low-pass filter. H1 represents the first high-pass filter. H2 represents the second high-pass filter. H3 represents the third high-pass filter. H4 represents the fourth high-pass filter. D1 represents the first downsampling. D2 represents the second downsampling. D3 represents the third downsampling. D4 represents the fourth downsampling. LL represents an approximate sub-image. LH represents a horizontal detail sub-image. HL represents a vertical detail sub-image. HH represents a diagonal detail sub-image.

DWT decomposes a signal into different scales using a set of basic functions (wavelets). In the decomposition stage, the signal undergoes separation into approximation coefficients and detail coefficients across various frequency ranges [33]. The approximation coefficients encapsulate the overall trend and low-frequency components of the signal, whereas the detail coefficients capture specific local details and high-frequency components. This decomposition allows for signal frequency characteristics to be analyzed at different scales, leading to a better understanding of the signal structure and variables. DWT finds wide application across signal processing, image processing, data compression, pattern recognition, and other fields [59]. In image processing, DWT is used for tasks such as image compression, denoising, and variable extraction [60]. The study selected the bior 1.3 wavelet basis function for decomposition, as illustrated in Figure 3. After DWT’s application to the original single-band images, four sub-images are obtained: approximate sub-image (LL), horizontal detail sub-image (LH), vertical detail sub-image (HL), and diagonal detail sub-image (HH). Transforming each single-band image of UAV multispectral imagery into wavelets resulted in 20 discrete wavelet variables being calculated.

### 2.4. Variable Selection and Machine Learning Algorithms

This research employed Recursive Feature Elimination (RFE) for variable selection. RFE progressively reduces the size of the variable set until a certain number of variables is reached and optimal performance is achieved [61]. This approach helps to reduce overfitting, improve model generalization, and identify the most critical variables for model performance [62]. RFE was combined with cross-validation to enhance the robustness and reliability of variable selection. In this study, cross-validated RFE was implemented using the Random Forest (RF) estimator.

Furthermore, four machine-learning algorithms were employed to develop models for predicting the SPAD values. These algorithms include Ridge Regression, RF, Support Vector Regression (SVR), and Back Propagation Neural Network (BPNN). Each algorithm possesses unique strengths in SPAD value prediction, addressing data complexity, and handling nonlinear (or linear) relationships to improve prediction accuracy and stability.

Ridge Regression is a linear regression method used to handle cases where the number of variables exceeds the number of samples or where there is multicollinearity among variables [63]. It controls model complexity by adding an L2 regularization term to prevent overfitting. Ridge Regression can effectively handle multicollinearity and noise in the dataset, improving model generalization.

RF is a type of ensemble learning algorithm that utilizes multiple decision trees. Each tree is built by randomly selecting subsets of variables and samples. The predictions from these trees are then combined through voting or averaging to produce the final prediction [64]. RF is known for its robustness and ability to generalize, effectively modeling complex relationships within high-dimensional datasets. These characteristics make it a suitable choice for predicting winter wheat SPAD values.

SVR is a regression technique derived from support vector machines (SVM), aiming to identify the maximum margin hyperplane within a high-dimensional variable space specifically for regression purposes. It is suitable for modeling nonlinear data and can handle nonlinear relationships by choosing appropriate kernel functions [65]. SVR can effectively model nonlinear relationships and exhibits robustness against outliers in predicting winter wheat SPAD values.

BPNN trains the model using the backpropagation algorithm to adjust weights to minimize the loss function continuously. BPNN is suitable for complex nonlinear problems and can learn complex patterns and variables in the data [66]. In predicting winter wheat SPAD values, BPNN can flexibly capture the dataset’s nonlinear relationships and complex patterns.

For parameter optimization, this research used a combination of cross-validation and grid search to optimize parameter combinations within a given parameter space, thereby improving model performance and generalization [67], leading to better results in predicting the SPAD values.

### 2.5. Dataset Splitting and Model Evaluation

The dataset was randomly divided into training and testing datasets in a ratio of 8:2, and K-fold (K = 5) cross-validation was employed to enhance the model’s generalization ability. The performance of the models was evaluated using four metrics: Coefficient of Determination (*R*^2^), Root Mean Square Error (*RMSE*), Relative Root Mean Square Error (*RRMSE*), and Ratio of Performance to Deviation (*RPD*). *RPD* aids in mitigating assessment biases arising from varying units or data scales [68].

The formulas of *R*^2^, *RMSE*, *RRMSE*, and *RPD* are presented in Equations (1)–(4):

(1) $$R^{2}=\frac{\sum {\left({\hat{y}}_{i}-\bar{y}\right)}^{2}}{\sum {\left(y_{i}-\bar{y}\right)}^{2}}$$

(2) $$RMSE=\sqrt{\frac{\sum_{i=1}^{n}{\left(\hat{y_{i}}-y_{i}\right)}^{2}}{n}}$$

(3) $$RRMSE=\frac{RMSE}{\bar{y}}$$

(4) $$RPD=\frac{SD}{RMSE}$$

where $y_{i}$ is the measured SPAD value of sample *i*; ${\hat{y}}_{i}$ is the predicted SPAD value of sample *i*; $\bar{y}$ is the mean SPAD value; *n* is the number of samples; SD is the standard deviation between predicted and measured SPAD values.

## 3. Results

### 3.1. RS Variable Selection

In the RFE variable selection process, this study employed learning curves derived from RFE to identify the appropriate number of RS variables. The RFE variable importance rankings were employed to determine the optimal set for subsequent modeling.

**Figure 4:**
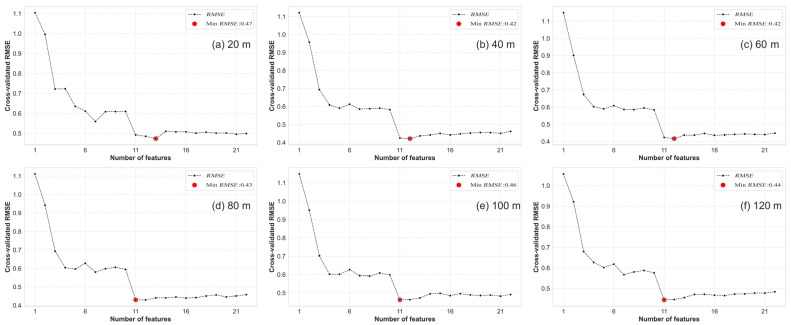
RFE learning curves based on VIs at different UAV flight altitudes: (**a**) 20 m VIs; (**b**) 40 m VIs; (**c**) 60 m VIs; (**d**) 80 m VIs; (**e**) 100 m VIs; (**f**) 120 m VIs.

Based on the RFE learning curves (Figure 4), the study identified the appropriate number of VIs at multiple UAV flight altitudes. At 20 m altitude (with a resolution of 1.06 cm/pixel), the appropriate number of VIs was identified as 13. At 40 m (with a resolution of 2.12 cm/pixel) and 60 m (with a resolution of 3.18 cm/pixel) altitudes, the appropriate number of VIs remained consistent at 12. At altitudes of 80 m (with a resolution of 4.23 cm/pixel), 100 m (with a resolution of 5.29 cm/pixel), and 120 m (with a resolution of 6.35 cm/pixel), the optimal number of VIs was 11. These optimal sets of VIs are listed in Table 3 and will serve as inputs for subsequent modeling. Across different altitudes, the optimal Vis selected for modeling include G, B, NIR, RVI, GRVI, TCARI/OSAVI, and WDRVI. Overall, the optimal number of selected VIs at different altitudes is roughly the same, but subtle differences exist in the specific VIs that were chosen. This suggests that VIs at different altitudes may exhibit slight variations in reflecting the growth status of winter wheat. Therefore, the modeling and analysis should use the appropriate VIs selected for different altitudes. This result further emphasizes the significance of screening RS variables at different altitudes to optimize the performance and accuracy of the model.

**Figure 5:**
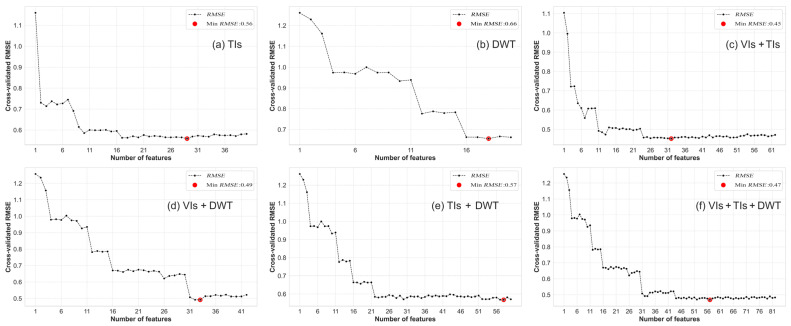
RFE learning curves under different variable combinations: (**a**) TIs; (**b**) DWT; (**c**) VIs + TIs; (**d**) VIs + DWT; (**e**) TIs + DWT; (**f**) VIs + TIs + DWT. Note: The RFE learning curve based on VIs is shown in Figure 4a.

The appropriate number of RS variables determined through RFE variable selection learning curves (Figure 4 and Figure 5) under different variable sets (VIs, TIs, DWT, VIs + TIs, VIs + DWT, TIs + DWT, and VIs + TIs + DWT) were found to be 13, 29, 18, 32, 33, 58, and 57, respectively. Subsequently, optimal RS variable sets for different variable combinations were determined based on the RFE variable importance ranking. In the TIs variable set, mean and correlation were selected as the optimal RS variables across different channels. Within the DWT set, LL and HH were chosen as the optimal RS variables across different channels. The selected RS variables in the VIs set are shown in Table 4. Details of the specific selected RS variables in the TIs and DWT sets can be found in Table 4. The specific lists of selected RS variables in the VIs + TIs and VIs + TIs + DWT sets are provided in Table 5. These results provide important clues for subsequent modeling and analysis, aiding in a deeper understanding of the relationship between the SPAD values and various RS variables.

### 3.2. Development and Validation of Winter Wheat Booting Stage SPAD Value Prediction Models at Different UAV Flight Altitudes

In this study, we first examined the performance of predicting SPAD for winter wheat by using UAV (DJI P4-Multispectral UAV) images at higher flight altitudes of 40 m (with a resolution of 2.12 cm/pixel), 60 m (with a resolution of 3.18 cm/pixel), 80 m (with a resolution of 4.23 cm/pixel), 100 m (with a resolution of 5.29 cm/pixel), and 120 m (with a resolution of 6.35 cm/pixel), through a comparison with the prediction performance using images at a baseline altitude of 20 m (with a resolution of 1.06 cm/pixel). Four different machine learning algorithms, including Ridge, RF, SVR, and BPNN, were employed in this study. In this objective, we only used VIs as predictor variables.

The performance of models based on VIs combined with multiple machine-learning algorithms varied significantly at different UAV flight altitudes (Table 6). For instance, the Ridge model performed best at 60 m altitude (with a resolution of 3.18 cm/pixel), achieving an *RPD* of 2.2435. The RF model showed optimal performance at 20 m altitude (with a resolution of 1.06 cm/pixel) with an *RPD* of 1.8232. Both SVR and BPNN models performed best at 40 m altitude (with a resolution of 2.12 cm/pixel), with *RPD* values of 2.0617 and 1.8388, respectively. Overall, the Ridge and SVR models exhibited superior accuracy in predicting winter wheat booting stage SPAD values at multiple UAV flight altitudes compared to RF and BPNN models. Particularly, the Ridge model developed at 60 m flight altitude (with a resolution of 3.18 cm/pixel) emerged as the optimal model for predicting the SPAD values based on VIs (with an *R*^2^ of 0.7821, *RMSE* of 1.4424, *RRMSE* of 0.0293, and *RPD* of 2.2435 on the test dataset). It is also noteworthy that at flight altitudes of 80 m (with a resolution of 4.23 cm/pixel) and 100 m (with a resolution of 5.29 cm/pixel), the Ridge model achieved *RPD* values of 2.1459 and 2.1545, respectively. At a flight altitude of 120 m (with a resolution of 6.35 cm/pixel), the SVR model achieved an *RPD* value of 2.0547.

Of particular note is that, at different flight altitudes, VIs can be employed with specific machine-learning algorithms to develop winter wheat booting stage SPAD value prediction models with a very good performance (*RPD* > 2.0). For example, at 120 m (with a resolution of 6.35 cm/pixel) altitude, despite the slightly lower performance of the RF and BPNN models (with *RPDs* of 1.6635 and 1.7720 on the test set, respectively), the Ridge and RF models still demonstrate an outstanding performance (with *RPDs* of 2.0237 and 2.0547 on the test set, respectively). This indicates that at a flight altitude of 120 m (with a resolution of 6.35 cm/pixel), UAV-based models combining VIs with certain machine learning methods can develop highly effective winter wheat booting stage SPAD value prediction models.

**Figure 6:**
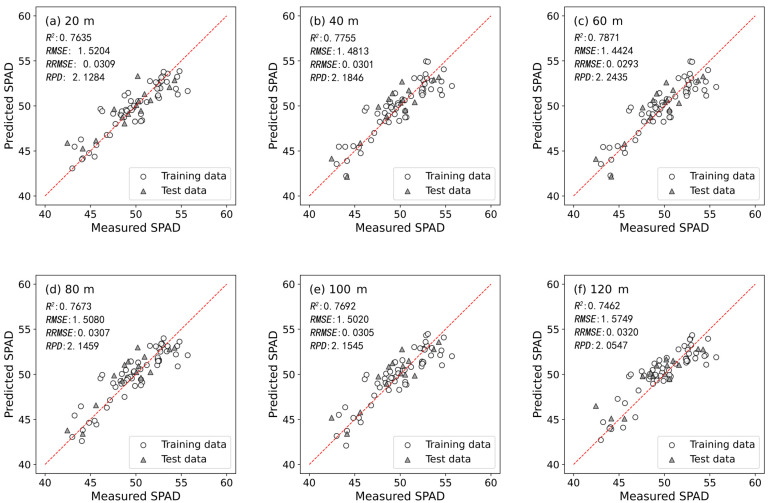
Scatter plots of all optimal models at multiple altitudes: (**a**) 20 m SVR; (**b**) 40 m Ridge; (**c**) 60 m Ridge; (**d**) 80 m Ridge; (**e**) 100 m Ridge; (**f**) 120 m SVR.

To further analyze the effectiveness of winter wheat booting stage SPAD value prediction models developed based on VIs at multiple flight altitudes, Figure 6 presents scatter plots comparing measured SPAD values with the predicted SPAD values for all optimal models at multiple flight altitudes. The small errors observed between the predicted and measured values highlight the effectiveness of predicting winter wheat booting stage SPAD values using the developed models.

### 3.3. Development and Validation of Winter Wheat Booting Stage SPAD Value Prediction Models under Different Variable Combinations

In this study, we investigated and compared the SPAD prediction performance for winter wheat between using individual types of predictor variable and using various combinations of predictor variables. Three different types of predictor variables were used in this study, encompassing VIs, TIs, and DWT variables. The same four machine learning algorithms were employed for the prediction. In this objective, we only used the images at an altitude of 20 m (with a resolution of 1.06 cm/pixel).

The performance differences in the winter wheat booting stage SPAD value prediction models developed based on different types of predictor variable were obvious (Table 7). For the VIs set, the winter wheat booting stage SPAD value prediction model developed using the SVR model exhibited the best performance (with an *R*^2^ of 0.7635, *RMSE* of 1.5204, *RRMSE* of 0.0309, and *RPD* of 2.1284 on the test dataset). Similarly, within the TIs set, the prediction model developed using the SVR model demonstrated the best performance (with *R*^2^ of 0.7812, *RMSE* of 1.4623, *RRMSE* of 0.0297, and *RPD* of 2.2130 on the test dataset). For the DWT set, the prediction model developed using RF achieved the best performance (with *R*^2^ of 0.7023, *RMSE* of 1.7057, *RRMSE* of 0.0347, and *RPD* of 1.8972 on the test dataset). Overall, when developing winter wheat booting stage SPAD value prediction models using a single variable set, the overall accuracy ranking is TIs > VIs > DWT.

When combining multiple variable sets, the winter wheat booting stage SPAD value prediction model developed using SVR in the VIs + TIs set exhibited the best performance (with an *R*^2^ of 0.8148, *RMSE* of 1.3455, *RRMSE* of 0.0274, and *RPD* of 2.4050) on the test dataset. For the VIs + DWT set, the model developed using SVR also demonstrated the best performance (with an *R*^2^ of 0.7940, *RMSE* of 1.4189, *RRMSE* of 0.0288, and *RPD* of 2.2807) on the test dataset. For the TIs + DWT set, the model developed using SVR also demonstrated the best performance (with an *R*^2^ of 0.7909, *RMSE* of 1.4294, *RRMSE* of 0.0291, and *RPD* of 2.2639) on the test dataset. Similarly, in the VIs + TIs + DWT set, the model developed using SVR also demonstrated the best performance (with an *R*^2^ of 0.8390, *RMSE* of 1.2544, *RRMSE* of 0.0255, and *RPD* of 2.5798) on the test dataset.

The overall accuracy of the winter wheat booting stage SPAD value prediction models developed using different variable sets follows the order: VIs + TIs + DWT > VIs + TIs > VIs + DWT > TIs + DWT > TIs > VIs > DWT. Models developed by combining multiple variable sets performed notably better than those developed using a single variable set.

**Figure 7:**
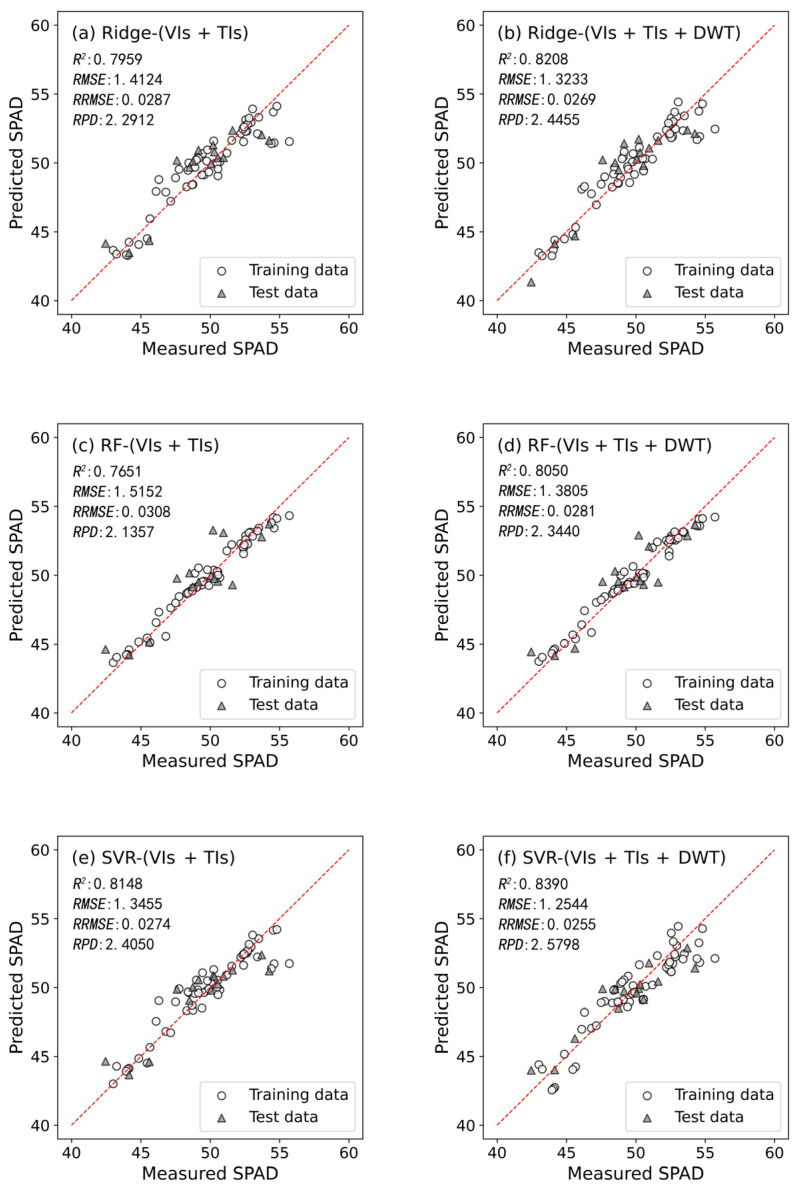

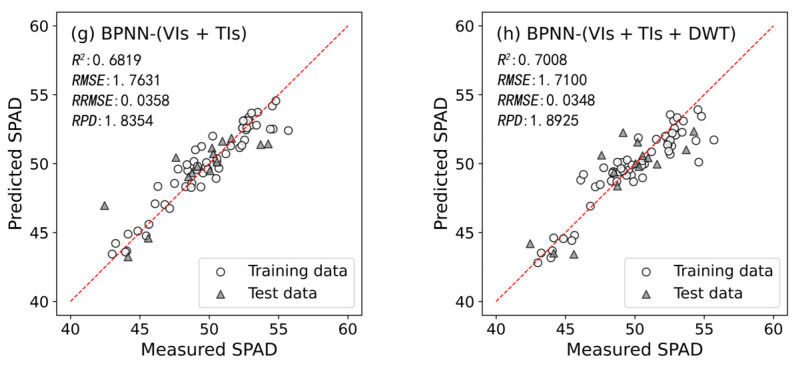
Scatter plots of all SPAD value prediction models under VIs + TIs and VIs + TIs + DWT combinations: (**a**) Ridge-(VIs + TIs); (**b**) Ridge-(VIs + TIs + DWT); (**c**) RF-(VIs + TIs); (**d**) RF-(VIs + TIs + DWT); (**e**) SVR-(VIs + TIs); (**f**) SVR-(VIs + TIs + DWT); (**g**) BPNN-(VIs + TIs); (**h**) BPNN-(VIs + TIs + DWT).

Furthermore, compared to the common use VIs + TIs set, the winter wheat booting stage SPAD value prediction model developed using the VIs + TIs + DWT set not only showed improved accuracy but also demonstrated a more stable performance (Figure 7). Under the VIs + TIs + DWT set, models developed using any machine learning algorithm performed excellently (except for the BPNN model, where Ridge, RF, and SVR models all had *R*^2^ values greater than 0.8). The winter wheat booting stage SPAD value prediction model developed using SVR in the VIs + TIs + DWT set, which achieved an *RPD* of 2.5798 on the test set, is particularly noteworthy. This model is the only one among the different developed models to achieve an *RPD* > 2.5 on the test dataset, demonstrating an excellent prediction performance. This further underscores the importance of combining the DWT set for winter wheat booting stage SPAD value prediction.

## 4. Discussion

### 4.1. Comparison of SPAD Value Prediction Accuracy at Varying UAV Flight Altitudes

In this study, we first examined the performance of predicting SPAD for winter wheat by using UAV (DJI P4-Multispectral UAV) images at higher flight altitudes of 40 m (with a resolution of 2.12 cm/pixel), 60 m (with a resolution of 3.18 cm/pixel), 80 m (with a resolution of 4.23 cm/pixel), 100 m (with a resolution of 5.29 cm/pixel), and 120 m (with a resolution of 6.35 cm/pixel) through a comparison with the prediction performance when using images at a baseline altitude of 20 m (with a resolution of 1.06 cm/pixel). Four different machine learning algorithms, including Ridge, RF, SVR, and BPNN, were employed in this study. In this objective, we only used VIs, which have been commonly used as predictor variables in similar previous studies. To enhance the reliability of the study results, winter wheat with various canopy structures was created by planting different varieties of winter wheat and applying different nitrogen fertilizer treatments within different plots (Figure 1 and Figure 2).

Within the flight altitude of 120 m (40 to 120 m, with a resolution from 2.12 to 6.35 cm/pixel), models for predicting winter wheat SPAD values during the booting stage were successfully developed using VIs combined with specific machine learning regressions (Ridge and SVR, using the flight altitude of 120 m (with a resolution of 6.35 cm/pixel) as an example), with *RPD* values exceeding 2.0. According to Viscarra Rossel et al. [68], models with *RPD* values exceeding 2.0 demonstrate a very good prediction performance, exceeding our expectations. Compared to the flight altitude of 20 m (with a resolution of 1.06 cm/pixel), the UAV at higher altitudes (40 to 120 m, with a resolution from 2.12 to 6.35 cm/pixel) were still able to capture clear spectral band reflectance values, facilitating the prediction of winter wheat SPAD values. Comparable findings were reported by Yang et al. [69] and Njane et al. [9], who suggested that VIs-based models are less affected by variations in UAV flight altitude within the range of 20–100 m (using a DJI P4-Multispectral UAV as an example, with a resolution from 1.06 to 5.29 cm/pixel).

The flight altitude of UAVs typically determines the flight duration, image pixel size, and the coverage area of fields [70]. While previous studies have suggested that a higher spatial resolution (lower flight altitude) allows for more detailed crop growth information and a more accurate prediction of crop parameters [10,11,12], particularly in terms if biomass and plant height [9], this conclusion is not contradictory to our findings. This is because images captured by UAVs are taken from above, and as UAV flight altitude increases, the height of the UAV and its coverage area cause plants farther from the UAV to appear smaller in the images, making it difficult to accurately predict morphological characteristics such as the volume (biomass) and height (plant height) of crops [71]. However, the prediction of SPAD values (physiological parameters of crops) using VIs at different flight altitudes differs significantly from predicting the morphological characteristics of crops using VIs at different UAV flight altitudes.

Moreover, a similar accuracy in predicting winter wheat SPAD values during the booting stage was achieved at higher flight altitudes (40 to 120 m, with a resolution from 2.12 to 6.35 cm/pixel) compared to the flight altitude of 20 m (with a resolution of 1.06 cm/pixel), indicating that higher UAV flight altitudes are a preferable option, facilitating the prediction of winter wheat SPAD values. This is because higher flight altitudes save time and battery during field missions, allowing for the collection of more plot images under limited battery conditions. Additionally, shorter flight activities reduce the likelihood of encountering lighting changes, avoiding the provision of misleading information about winter wheat due to images obtained under variable illumination.

### 4.2. Influence of Multiple Variable Sets on Winter Wheat SPAD Value Prediction during the Booting Stage

In this study, we investigated and compared the SPAD prediction performance for winter wheat when using individual types of predictor variable and using various combinations of predictor variables. Three different types of predictor variables were used in this study, encompassing VIs, TIs, and DWT variables. The same four machine learning algorithms (Ridge, RF, SVR, and BPNN) were employed for the prediction. In this objective, we only used the images at an altitude of 20 m (with a resolution of 1.06 cm/pixel).

The differences in model performance based on different variable sets were significant. Generally, when only one variable set was used to develop winter wheat SPAD value prediction models, the overall accuracy was as follows: TIs > VIs > DWT. This study found that models developed with the TIs set achieved higher accuracy in predicting winter wheat SPAD values during the booting stage than the VIs commonly used in previous studies. This may be because, under different nitrogen fertilizer treatments, some plots still had small winter wheat plants with more exposed soil. This condition potentially disrupted the canopy spectra’s responsiveness to SPAD value characteristics. TIs are sensitive to boundaries between soil and green plants [72], and accordingly, TIs (especially those under the R channel) demonstrated a stronger correlation with winter wheat SPAD values.

Although the accuracy of DWT in predicting winter wheat SPAD values slightly lagged behind that of TIs and VIs, acceptable prediction models could still be developed. The LL, HH, HL, and LH channels under different bands showed some degree of correlation with winter wheat SPAD values. This is because DWT effectively separates useful information from weak information, thereby utilizing existing information [36], which is a key rationale for introducing DWT in this study.

The overall accuracy of winter wheat SPAD value prediction models developed with different variable combinations was as follows: VIs + TIs + DWT > VIs + TIs > VIs + DWT > TIs + DWT > TIs > VIs > DWT. Models combining multiple variable sets performed significantly better than models developed with a single variable set. Although the accuracy of predicting winter wheat SPAD values using the VIs + TIs set was higher than that of using any single variable set alone, the overall improvement in accuracy was not significant. This may be because these two variable sets are already closely related to SPAD values. Therefore, their combination did not produce particularly significant synergistic effects [73].

Furthermore, compared to the VIs + TIs set, models developed with the VIs + TIs + DWT set not only showed improved accuracy but also demonstrated a more stable performance in predicting winter wheat SPAD values. The main reason for this may be that the VIs + TIs + DWT set combines the spectral (VIs), frequency (DWT), and spatial information (TIs) of multispectral images, compensating for the shortcomings of using only spectral and spatial variables [74]. Under the VIs + TIs + DWT set, prediction models developed with any machine learning algorithm performed excellently.

Notably, under the VIs + TIs + DWT set, the prediction model developed with SVR achieved an *RPD* of 2.5798 on the test set. This model was the only one built under different variable combinations with an *RPD* exceeding 2.5 on the test set, demonstrating an excellent prediction performance [68]. This further underscores the importance of combining the DWT set for predicting winter wheat SPAD values. Combining VIs, TIs, and DWT can achieve a better prediction of winter wheat SPAD values during the booting stage, serving as an alternative to advanced cameras or longer lenses.

### 4.3. Performance Comparison of Four Machine Learning Models

Under different UAV flight altitudes, VIs combined with specific machine learning models were able to develop highly accurate models for predicting winter wheat SPAD values. Ridge and SVR models demonstrated distinct advantages over RF and BPNN models at different altitudes, exhibiting notable stability and accuracy. Across different variable sets (VIs, TIs, VIs + TIs, VIs + DWT, TIs + DWT, VIs + TIs + DWT), models developed by SVR performed best in predicting the SPAD values during the booting stage. This suggests that SVR models are more suitable for predicting winter wheat SPAD values during the booting stage. This may be attributed to the objective of the SVR optimization problem, which aims to minimize training errors while maximizing the margin, resulting in models that are typically globally optimal [75] and enabling SVR to better generalize to new data in some cases.

Some studies have suggested that RF models outperform SVR models in predicting crop parameters. For instance, Osco et al. [76] found that RF models could more accurately predict leaf nitrogen content (LNC) in maize compared to SVR models. Likewise, Zha et al. [77] demonstrated that RF models outperformed SVR and Artificial Neural Network models in estimating the rice nitrogen nutrition index (NNI). However, given the excellent performance of SVR models in this study, especially their achieving the highest accuracy in VIs, TIs, VIs + TIs, and VIs + TIs + DWT sets, the superiority of RF models may require further research verification.

Additionally, in this research, the optimal number of input variables for the models was identified using the RFE learning curve. It was observed that increasing the number of input variables beyond a specific point did not improve accuracy; instead, it led to a decrease in accuracy. This finding underscores the importance of identifying the optimal number of input variables to reduce information redundancy, ultimately enhancing model efficiency and prediction accuracy.

### 4.4. Limitations and Future Directions

We will actually fly the UAV to obtain images at 40 m (with a resolution of 2.12 cm/pixel), 60 m (with a resolution of 3.18 cm/pixel), 80 m (with a resolution of 4.23 cm/pixel), 100 m (with a resolution of 5.29 cm/pixel), and 120 m (with a resolution of 6.35 cm/pixel) in future research. In this way, we can obtain raw UAV images at different flight altitudes, rather than resampled images. This will reduce uncertainties resulted from the use of different resampling algorithms. In addition, it can provide the time used for monitoring the field at different altitudes as evidence when discussing the effectiveness of flying at elevated altitudes.

Additionally, given that previous studies have highlighted the lower accuracy in predicting SPAD values during the winter wheat booting stage compared to other growth stages, this study concentrated solely on this stage, with plans for future research to encompass additional growth stages. Moreover, this study relied on data from a single year of experimentation, emphasizing the need for further validation in subsequent research endeavors.

Furthermore, neglecting the significant vertical gradients in SPAD values and treating the canopy as a uniform plane can compromise the robustness of canopy RS and diminish its practical applicability, as suggested by earlier studies [78]. Future research will consider the issue of the uneven vertical distribution of SPAD values and use advanced sensors such as LiDAR to obtain more winter wheat SPAD value-related characteristics to address these issues.

## 5. Conclusions

This study demonstrates that VIs combined with specific machine learning algorithms can achieve similar accuracy in predicting winter wheat SPAD values during the booting stage at higher flight altitudes (40 to 120 m, using a DJI P4-Multispectral drone as an example, with a resolution from 2.12 to 6.35 cm/pixel) to the flight altitude of 20 m (with a resolution of 1.06 cm/pixel). The result suggests that the flight altitude of 120 m (with a resolution of 6.35 cm/pixel) is an alternative that can achieve comparable results to a lower flight altitude at 20 m (with a resolution of 1.06 cm/pixel) with a balanced tradeoff between accuracy and efficiency. This allows for the collection of more field images under limited battery conditions. It also avoids providing misleading information about winter wheat due to images being obtained under variable illumination, thereby facilitating the large-scale monitoring of winter wheat in actual agricultural production.

The overall accuracy of winter wheat SPAD value prediction models developed with different variable sets was VIs + TIs + DWT > VIs + TIs > VIs + DWT > TIs + DWT > TIs > VIs > DWT. Models developed with the TIs set achieved a higher accuracy in predicting winter wheat SPAD values than the VIs commonly used in previous studies, presenting a promising alternative approach. Additionally, although the accuracy of DWT in predicting winter wheat SPAD values slightly lagged behind that of TIs and VIs, acceptable prediction models could still be developed.

Models combining multiple variable sets performed significantly better than models developed with a single variable set. Furthermore, compared to the commonly used VIs + TIs set in previous studies, the VIs + TIs + DWT set used in this study combined the spectral (VIs), frequency (DWT), and spatial (TIs) information of multispectral images. This combination compensates for the limitations of solely using spectral and spatial variables. The resulting winter wheat SPAD value prediction models not only showed improved accuracy but also demonstrated a more stable performance. This provides more meaningful technical support for the RS prediction of winter wheat SPAD values, facilitating more sophisticated field management practices in precision agriculture.

## Figures and Tables

**Table 1:** Specifications of the SPAD-502Plus handheld chlorophyll meter.

Main Specifications	Specification Parameters
Measurement principle	The difference in optical density at two wavelengths *
Measurement range	0 to 99.9 SPAD units
Sample area	2 × 3 mm
Measurement time	Approximately 2 s per sample
Sample thickness	Maximum 1.2 mm
Accuracy	±1.0 SPAD units
Operating temperature	0–50 °C, relative humidity up to 85% (at 35 °C), no condensation

* Note: The SPAD-502Plus measures the absorbance of chlorophyll at two wavelengths (650 nm and 940 nm) to estimate chlorophyll content.

**Table 2:** Twenty-two VIs were used in this study for predicting SPAD values during the winter wheat booting stage.

Vis	Formulation	References
R, G, B, RE, NIR	/	/
RVI	NIR/R	[43]
GCI	(NIR/G) − 1	[44]
RECI	(NIR/RE) − 1	[44]
TCARI	3 × [(RE − R) − 0.2 × (RE − G) × (RE/R)]	[45]
NDVI	(NIR − R)/(NIR + R)	[46]
GNDVI	(NIR − G)/(NIR + G)	[47]
GRVI	(G − R)/(G + R)	[43]
NDRE	(NIR − RE)/(NIR + RE)	[48]
NDREI	(RE − G)/(RE + G)	[49]
SCCCI	NDRE/NDVI	[50]
EVI	2.5 × (NIR − R)/(1 + NIR − 2.4 × R)	[51]
EVI2	2.5 × (NIR − R)/(NIR + 2.4 × R + 1)	[52]
OSAVI	(NIR − R)/(NIR − R + L) (L = 0.16)	[53]
MCARI	[(RE − R) − 0.2 × (RE − G)] × (RE/R)	[54]
TCARI/OSAVI	TCARI/OSAVI	[55]
MCARI/OSAVI	MCARI/OSAVI	[54]
WDRVI	(a × NIR − R)/(a × NIR + R) (a = 0.12)	[55]

**Table 3:** Optimal VIs selected at multiple UAV flight altitudes.

VIs	RFE					
	20 m	40 m	60 m	80 m	100 m	120 m
R		√	√			
G	√	√	√	√	√	√
B	√	√	√	√	√	√
NIR	√	√	√	√	√	√
Rededge	√	√	√	√	√	√
RVI	√	√	√	√	√	√
GCI		√				
RECI				√		√
NDVI						
GNDVI	√		√			
GRVI	√	√	√	√	√	√
NDRE	√					
NDREI	√				√	√
SCCCI		√	√	√	√	√
OSAVI		√	√	√	√	√
EVI						
EVI2						
MCARI	√					
TCARI						
MCARI/OSAVI	√					
TCARI/OSAVI	√	√	√	√	√	√
WDRVI	√	√	√	√	√	√

Note: “√” refers to the optimal variables that were used to develop the predictive models.

**Table 4:** Optimal sets of RS variables selected from TIs set and DWT set.

Variable Set	TIs	R	G	B	NIR	Rededge
TIs	mean	√	√	√	√	√
	variance	√	√	√		√
	homogeneity	√	√	√		√
	contrast	√	√		√	
	dissimilarity	√	√	√		
	entropy				√	
	second moment		√	√	√	√
	correlation	√	√	√	√	√
DWT	LL	√	√	√	√	√
	LH	√		√	√	√
	HL	√		√	√	√
	HH	√	√	√	√	√

Note: The optimal variable set selected from the VIs set is shown in Table 4. “√” refers to the optimal variables that were used to develop the predictive models.

**Table 5:** Optimal sets of RS variables selected from the VIs + TIs set, VIs + DWT set, TIs + DWT set, and VIs + TIs + DWT set.

Variable Set	Optimal Variables
VIs + TIs	R, NIR, RE, RVI, GCI, RECI, NDRE, SCCCI, OSAVI, EVI, EVI2, TCARI, MCARI/OSAVI, TCARI/OSAVI, WDRVI, R-mean, R-variance, R-homogeneity, R-dissimilarity, R-correlation, G-mean, G-secondmoment, G-correlation, B-homogeneity, B-contrast, B-dissimilarity, NIR-mean, NIR-homogeneity, NIR-contrast, RE-mean, RE-secondmoment, RE-correlation
VIs + DWT	R, G, NIR, RE, RVI, GCI, GNDVI, NDRE, NDREI, SCCCI, EVI2, MCARI, TCARI, MCARI/OSAVI, WDRVI, R_HH, R_HL, R_LH, R_LL, G_HH, G_LH, G_LL, B_HL, B_LH, B_LL, NIR_HH, NIR_HL, NIR_LH, NIR_LL, RE_HH, RE_HL, RE_LH, RE_LL
TIs + DWT	R-mean, R-variance, R-homogeneity, R-contrast, R-dissimilarity, R-entropy, R-correlation, G-mean, G-variance, G-homogeneity, G-contrast, G-dissimilarity, G-entropy, G-secondmoment, G-correlation, B-mean, B-variance, B-homogeneity, B-contrast, B-dissimilarity, B-entropy, B-secondmoment, NIR-mean, NIR-variance, NIR-homogeneity, NIR-contrast, NIR-dissimilarity, NIR-entropy, NIR-secondmoment, NIR-correlation, RE-mean, RE-variance, RE-homogeneity, RE-contrast, RE-dissimilarity, RE-entropy, RE-secondmoment, RE-correlation, R_HH, R_HL, R_LH, R_LL, G_HH, G_HL, G_LH, G_LL, B_HH, B_HL, B_LH, B_LL, NIR_HH, NIR_HL, NIR_LH, NIR_LL, RE_HH, RE_HL, RE_LH, RE_LL
VIs + TIs + DWT	R, NIR, RE, RVI, GCI, GNDVI, NDRE, NDREI, SCCCI, TCARI, MCARI/OSAVI, WDRVI, R-mean, R-variance, R-homogeneity, R-contrast, R-entropy, R-correlation, G-mean, G-contrast, G-correlation, B-mean, B-variance, B-homogeneity, B-contrast, B-dissimilarity, B-entropy, B-secondmoment, NIR-mean, NIR-variance, NIR-dissimilarity, NIR-entropy, NIR-secondmoment, NIR-correlation, RE-mean, RE-variance, RE-homogeneity, RE-contrast, RE-dissimilarity, RE-entropy, RE-secondmoment, RE-correlation, R_HH, R_HL, R_LH, R_LL, G_LL, B_HH, B_HL, B_LH, B_LL, NIR_HH, NIR_HL, NIR_LL, RE_HH, RE_LH, RE_LL

**Table 6:** Comparison of modeling accuracy of VIs at different UAV flight altitudes.

Altitude	Model	Train				Test			
		*R* ^2^	*RMSE*	*RRMSE*	*RPD*	*R* ^2^	*RMSE*	*RRMSE*	*RPD*
20 m	Ridge	0.7400	1.6298	0.0327	1.9787	0.7092	1.6858	0.0343	1.9196
	RF	0.9364	0.6119	0.0123	5.2705	0.6777	1.7750	0.0361	1.8232
	SVR	0.8038	1.4159	0.0284	2.2777	0.7635	1.5204	0.0309	2.1284
	BPNN	0.6640	1.8529	0.0371	1.7405	0.6609	1.8206	0.0370	1.7775
40 m	Ridge	0.7994	1.4317	0.0287	2.2526	0.7755	1.4813	0.0301	2.1864
	RF	0.9628	0.6163	0.0123	5.2328	0.6098	1.9530	0.0397	1.6570
	SVR	0.7921	1.4576	0.0292	2.2125	0.7479	1.5696	0.0319	2.0617
	BPNN	0.6920	1.7741	0.0355	1.8178	0.6831	1.7599	0.0358	1.8388
60 m	Ridge	0.7961	1.4433	0.0289	2.2345	0.7871	1.4424	0.0293	2.2435
	RF	0.9735	0.5208	0.0104	6.1920	0.6131	1.9447	0.0395	1.6640
	SVR	0.7722	1.5256	0.0306	2.1139	0.7203	1.6533	0.0336	1.9574
	BPNN	0.7010	1.7480	0.0350	1.8450	0.6817	1.7637	0.0359	1.8348
80 m	Ridge	0.8060	1.4079	0.0282	2.2906	0.7673	1.5080	0.0307	2.1459
	RF	0.9606	0.6344	0.0127	5.0837	0.5924	1.9961	0.0406	1.6212
	SVR	0.7413	1.6257	0.0326	1.9837	0.7078	1.6900	0.0344	1.9148
	BPNN	0.7006	1.7491	0.0350	1.8438	0.6773	1.7760	0.0361	1.8221
100 m	Ridge	0.7884	1.4704	0.0295	2.1932	0.7692	1.5020	0.0305	2.1545
	RF	0.9605	0.6355	0.0127	5.0746	0.6201	1.9270	0.0392	1.6793
	SVR	0.7944	1.4493	0.0290	2.2251	0.7350	1.6094	0.0327	2.0107
	BPNN	0.6922	1.7735	0.0355	1.8185	0.6617	1.8185	0.0370	1.7795
120 m	Ridge	0.7797	1.5002	0.0301	2.1497	0.7384	1.5991	0.0325	2.0237
	RF	0.9598	0.6410	0.0128	5.0315	0.6128	1.9453	0.0395	1.6635
	SVR	0.7614	1.5613	0.0313	2.0656	0.7462	1.5749	0.0320	2.0547
	BPNN	0.7043	1.7381	0.0348	1.8554	0.6588	1.8262	0.0371	1.7720

**Table 7:** Comparison of modeling accuracy under different variable combinations.

Variable Set	Model	Train				Test			
		*R* ^2^	*RMSE*	*RRMSE*	*RPD*	*R* ^2^	*RMSE*	*RRMSE*	*RPD*
VIs	Ridge	0.7400	1.6298	0.0327	1.9787	0.7092	1.6858	0.0343	1.9196
	RF	0.9364	0.6119	0.0123	5.2705	0.6777	1.7750	0.0361	1.8232
	SVR	0.8038	1.4159	0.0284	2.2777	0.7635	1.5204	0.0309	2.1284
	BPNN	0.6640	1.8529	0.0371	1.7405	0.6609	1.8206	0.0370	1.7775
TIs	Ridge	0.9160	0.9263	0.0186	3.4816	0.7212	1.6507	0.0336	1.9603
	RF	0.9576	0.6580	0.0132	4.9015	0.6866	1.7502	0.0356	1.8489
	SVR	0.8684	1.1594	0.0232	2.7816	0.7812	1.4623	0.0297	2.2130
	BPNN	0.8889	1.0654	0.0213	3.0269	0.6265	1.9106	0.0388	1.6938
DWT	Ridge	0.6948	1.7658	0.0354	1.8263	0.6563	1.8327	0.0373	1.7657
	RF	0.9502	0.7135	0.0143	4.5198	0.7023	1.7057	0.0347	1.8972
	SVR	0.6838	1.7975	0.0360	1.7941	0.6744	1.7840	0.0363	1.8139
	BPNN	0.7864	1.4772	0.0296	2.1831	0.5076	2.1937	0.0446	1.4752
VIs + DWT	Ridge	0.7372	1.6388	0.0328	1.9679	0.5324	2.1377	0.0435	1.5138
	RF	0.9679	0.5724	0.0115	5.6345	0.7296	1.6258	0.0331	1.9904
	SVR	0.7899	1.4651	0.0294	2.2012	0.7940	1.4189	0.0288	2.2807
	BPNN	0.7399	1.6302	0.0327	1.9783	0.4418	2.3357	0.0475	1.3855
TIs + DWT	Ridge	0.8797	1.1102	0.0222	2.9048	0.7824	1.4582	0.0296	2.2191
	RF	0.9570	0.6625	0.0133	4.8679	0.7151	1.6688	0.0339	1.9392
	SVR	0.9008	1.0068	0.0202	3.2031	0.7909	1.4294	0.0291	2.2639
	BPNN	0.8403	1.2773	0.0256	2.5248	0.6043	1.9665	0.0400	1.6455
VIs + TIs	Ridge	0.8651	1.1742	0.0235	2.7465	0.7959	1.4124	0.0287	2.2919
	RF	0.9687	0.5658	0.0113	5.7003	0.7651	1.5152	0.0308	2.1357
	SVR	0.8816	1.1000	0.0220	2.9319	0.8148	1.3455	0.0274	2.4050
	BPNN	0.8996	1.0126	0.0203	3.1847	0.6819	1.7631	0.0358	1.8354
VIs + TIs + DWT	Ridge	0.8959	1.0315	0.0207	3.1265	0.8208	1.3233	0.0269	2.4455
	RF	0.9664	0.5860	0.0117	5.5029	0.8050	1.3805	0.0281	2.3440
	SVR	0.8504	1.2364	0.0248	2.6083	0.8390	1.2544	0.0255	2.5798
	BPNN	0.8316	1.3117	0.0263	2.4586	0.7025	1.7051	0.0347	1.8978

## Data Availability

The data are available from the authors upon reasonable request as the data need further use.
